# Time Efficient 3D Radial UTE Sampling with Fully Automatic Delay Compensation on a Clinical 3T MR Scanner

**DOI:** 10.1371/journal.pone.0150371

**Published:** 2016-03-14

**Authors:** Karl-Heinz Herrmann, Martin Krämer, Jürgen R. Reichenbach

**Affiliations:** Medical Physics Group, Institute of Diagnostic and Interventional Radiology, Jena University Hospital, Friedrich-Schiller-University Jena, Jena, Germany; University of Chicago, UNITED STATES

## Abstract

This work’s aim was to minimize the acquisition time of a radial 3D ultra-short echo-time (UTE) sequence and to provide fully automated, gradient delay compensated, and therefore artifact free, reconstruction. The radial 3D UTE sequence (echo time 60 μs) was implemented as single echo acquisition with center-out readouts and improved time efficient spoiling on a clinical 3T scanner without hardware modifications. To assess the sequence parameter dependent gradient delays each acquisition contained a quick calibration scan and utilized the phase of the readouts to detect the actual *k*-space center. This calibration scan does not require any user interaction. To evaluate the robustness of this automatic delay estimation phantom experiments were performed and 19 *in vivo* imaging data of the head, tibial cortical bone, feet and lung were acquired from 6 volunteers. As clinical application of this fast 3D UTE acquisition single breath-hold lung imaging is demonstrated. The proposed sequence allowed very short repetition times (TR~1ms), thus reducing total acquisition time. The proposed, fully automated *k*-phase based gradient delay calibration resulted in accurate delay estimations (difference to manually determined optimal delay −0.13 ± 0.45 μs) and allowed unsupervised reconstruction of high quality images for both phantom and *in vivo* data. The employed fast spoiling scheme efficiently suppressed artifacts caused by incorrectly refocused echoes. The sequence proved to be quite insensitive to motion, flow and susceptibility artifacts and provides oversampling protection against aliasing foldovers in all directions. Due to the short TR, acquisition times are attractive for a wide range of clinical applications. For short T2* mapping this sequence provides free choice of the second TE, usually within less scan time as a comparable dual echo UTE sequence.

## Introduction

Magnetic resonance imaging (MRI) of materials and tissues with very short T_2_ relaxation times is a challenging task, which requires fast signal acquisition starting immediately after the RF excitation. To meet this challenge *ultra-short echo time* (UTE) imaging sequences have been successfully applied by using slice selective 2D radial acquisition [1, 2] to image brain tissue [3], cortical bone [4, 5, 6] and tendons [4, 7]. An acquisition-weighted stack of spirals sequence has been recently proposed for high resolution 3D UTE imaging [8]. Disregarding zero echo time techniques [9, 10], the shortest possible echo times can only be achieved by using 3D radial center-out acquisitions with no slice selection, slice rewinding or phase encoding gradients. Such 3D radial UTE acquisitions can be applied to a wide range of organs like knees and ankles [11], cortical bone [12] or for dental [13, 14] and cardiovascular imaging [15]. Besides providing the shortest possible echo times, the 3D radial center-out trajectory is also very robust against motion and flow artifacts due to its inherent flow compensation.

One major limitation and possible advantage of 3D radial acquisitions is the strong oversampling of inner *k*-space which result from fulfilling Nyquist sampling in the outer *k*-space parts. In particular, full Nyquist sampling requires π times the number of readouts compared to 3D Cartesian sampling which increases the total acquisition time and creates large raw data sets that can be challenging to handle by the reconstruction system. Image reconstruction from radially acquired *k*-space data is further complicated by the mandatory correction of gradient delays and eddy current effects. The actual gradient delays depend on scanner hardware and scan parameters and have to be compensated in order to obtain artifact free images [16, 17]. Due to this acquisition specific delay compensation the sampling density has to be calculated for each individual data set, and 3D radial gridding reconstruction [18] has to be performed for the entire 3D image matrix at once while taking into account all input data, thus increasing the computational demands substantially compared to Cartesian or 2D UTE approaches. Additionally we aim towards a fully automatic reconstruction and a delay calibration which does not require any kind of user interactions or planning.

One possibility to reduce the scan time is to exploit the significant time saving potential of center-out trajectories, thereby shortening the repetition time (TR) and, consequently, total acquisition time (TA). Many 3D radial UTE implementations use center-out trajectories in combination with full rewinding of the readout and an additional constant spoiler [11, 19] to achieve predictable RF spoiling. However, this approach leads to low scan efficiency as more time is spent for gradient rewinding and spoiling than for data acquisition. Alternative randomized spoiling regimes, which do not reach a steady state, are feasible [20] but still employ additional spoiler gradients which increase the minimum TR.

In this work, we present an optimized UTE 3D radial sequence, which employs time efficient sampling and spoiling to provide shortest possible TR. The sequence also includes a quick and automatic calibration scan for automatic gradient delay correction, which is performed prior to each scan. To demonstrate the robustness and the application spectrum of the developed fast UTE 3D sequence a variety of different body parts and organs were imaged with different scan parameters. To particularly utilize the shorter TR and scan time single breath-hold lung images were acquired in volunteers.

## Material and Methods

All measurements were performed on a clinical 3T whole body system (TIM Trio, Siemens Health Care, Erlangen, Germany) with standard hard- and software configuration and using vendor supplied coils. The installed scanner software version (VB17) did not include a UTE implementation which could have been used as a reference. Consequently, to provide reference images, a dual echo UTE sequence with full rewinding and spoiling [19, 21, 22] and the new time optimized single echo UTE sequence were both implemented.

**Fig 1:**
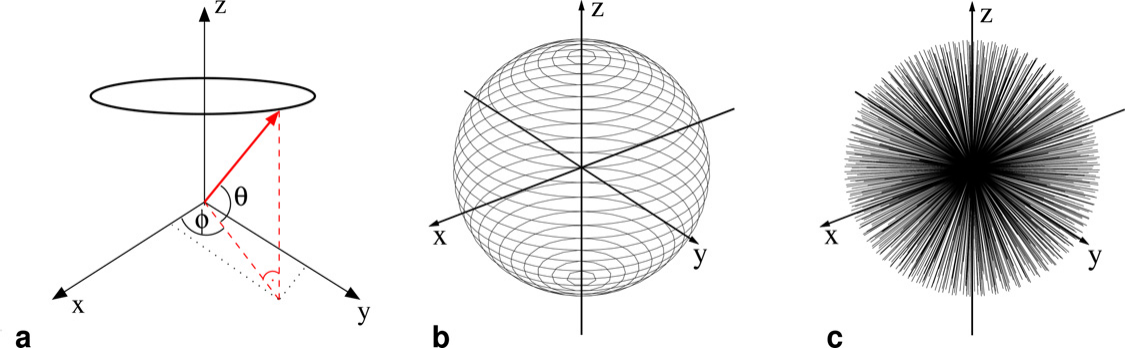
**Acquisition Trajectories:** (a) Definition of the coordinate system for the radial readout spokes. (b) Illustration of circles of constant latitude on a spherical surface of radius $r=\frac{M}{2}\;\Delta k$. Each readout spoke points to a different end position on these circles and the endpoints are evenly distributed on the circles. (c) Resulting 3D distribution of measured spokes.

**3D radial *k*-space trajectories.** Geometrically, the readout sampling points of the two sequences follow concentric cone planes [23, 24]. The Nyquist sampling theorem is fulfilled by using equidistant spacing Δ*k* = 1/*FoV* of the sampling points on the spherical surface (Fig 1). The circles of constant latitude on which the endpoints of the trajectories are evenly distributed are illustrated in Fig 1B, the resulting homogeneous distribution of the actual readout trajectories is shown in Fig 1C.

**Fig 2:**
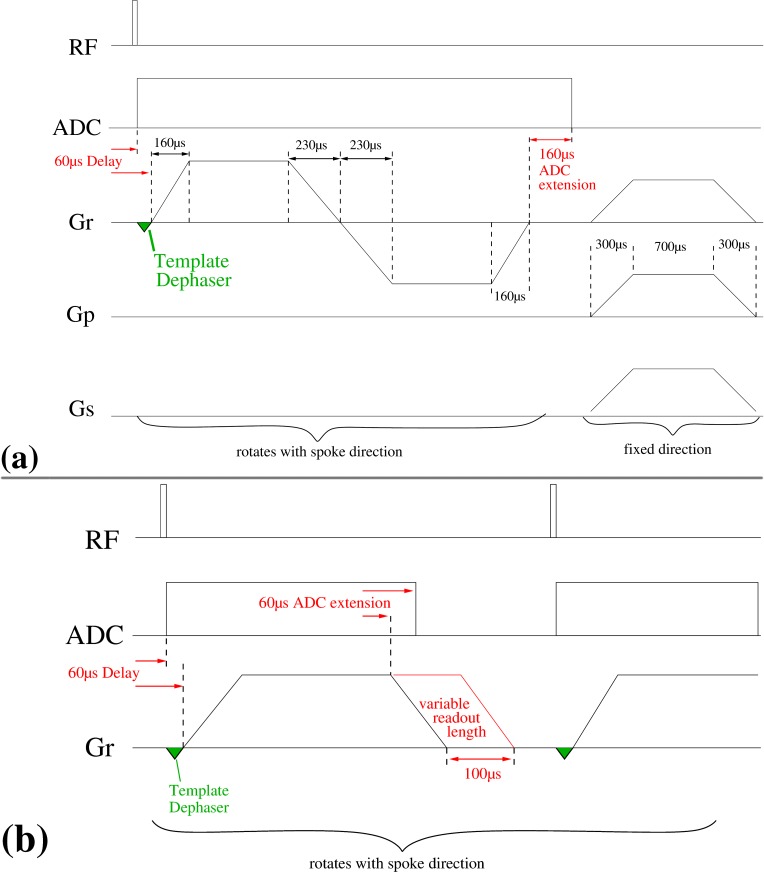
**Sequence Diagrams:** Schematic sequence diagrams of the dual echo (a) and the optimized single echo (b) UTE sequence. In both sequences the template dephaser gradient is only applied during the calibration scans; during image data acquisition the amplitude is set to zero. The readout gradient is distributed across all three physical gradient axes x, y and z according to the particular spoke direction (θ, ϕ) (see Fig 1 for the direction scheme). In (a), spoiler gradients are not rotated. However, for the single echo sequence (b) spoiling is affected by extending the readout gradient up to 100 μs beyond the nominal data acquisition and, consequently, the spoiling gradient rotates with the readout direction. This spoiler part of the readout gradient is linearly modified during each cone acquisition, starting from maximum duration (ϕ = 0°) to minimum duration (ϕ = 359°).

**Dual echo radial UTE acquisition.** The dual echo radial sequence acquires two echoes: the FID on the center-out trajectory and the gradient recalled echo during the center-in trajectory. The corresponding sequence diagram is shown in Fig 2A. To obtain shortest possible echo times, the analog to digital converter (ADC) was switched on immediately after a short RF pulse of 20 μs duration. As data acquired during the first 50–100 μs may, however, be degraded by artifacts due to coil tuning and digital filtering of the ADC [10], faulty data points were discarded before re-gridding the data.

For the standard receive coils a delay of 60 μs between the start of the ADC and ramping the readout gradients ensured sufficient time for the hardware to switch from the transmit to the receive state and to provide a stable, artifact free signal. To keep the acquisition time at a minimum, ramp sampling was performed. The ADC was extended to acquire data for additional 160 μs.

RF spoiling was used in addition to applying constant spoiler gradients on all three gradient axes (ramp up/plateau/ramp down = 300 μs/700 μs/300 μs with an amplitude of 22 mT/m). This spoiling scheme provides *constant spoiling moments* for each TR period *regardless* of the *spoke orientation* [11].

**Time optimized single echo radial UTE acquisition.** The dual echo acquisition was modified into a single echo acquisition consisting only of a center-out trajectory. Without rewinding the readout gradient, however, the total gradient moment during each TR period is not constant and depends on the readout direction, making spoiling effects difficult to predict. To minimize TR, no additional constant spoiling gradient was used. Instead, each readout gradient’s plateau time was extended by up to 100 μs beyond the nominal plateau time (Fig 2B) to provide additional spoiling moment in the direction of the current spoke. As the individual readouts are sampled sequentially, acquiring one cone plane after the other, this scheme leads to a quasi-constant spoiling moment for reasonably large matrix sizes (> = 128), especially as the spoiling moment along the z-direction varies only slowly compared to the typical T_2_ times of tissue. To further pseudo randomize the spoiling moment of spokes [20] within a given cone the extension of the readout gradient was linearly decreased from the maximum duration of 100 μs at ϕ = 0° to the minimum additional gradient duration of 0 μs at ϕ = 359°.

### Compensating Gradient Errors

Radial trajectories are sensitive to even smallest imperfections in the gradient and sampling hardware. Such imperfections may originate from a wide range of sources and depend on the vendor, the system type and even the specific MRI scanner used [25, 26, 27]. For a specific system, gradients may show constant, but different delays for each physical *x*-, *y*- and *z*-axis [16]. In addition, the different timing grids for RF pulses, data sampling and gradient activities can cause jitter between sampling and gradient activity that depends on sequence parameters like bandwidth, field of view and matrix size [17]. The digitally filtered ADC data from each coil channel may be a further source of artifacts as well as time shifts. Hardware delays and, in particular, scan parameter dependent timing jitter may cause offsets between the nominal start of gradient activity and ADC sampling that are difficult to predict. To re-grid the data point at *k*-space center and on the ramp correctly these time shifts must be known very precisely (<1 μs) to avoid image artifacts during reconstruction.

#### Delay calibration scans

**Fig 3:**
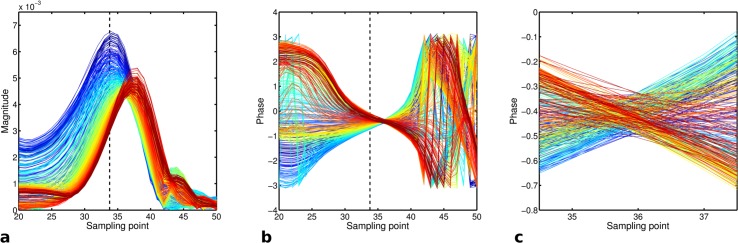
Gradient delay calibration using *k*-phase: Shown are 45 spokes of the measured calibration data from the x-y plane to illustrate the concept. The magnitude is shown in (a), the phase in (b) and an enlarged central part of (b) is shown in (c). The colors characterize the spoke index using a continuous jet color map where blue is the first and red the last index. The dashed black line marks the expected position of the *k*-space center.

To reliably estimate these system delays for each data set both sequences employed a short (dual echo: < 5 s; single echo: < 1.5 s) template scan which acquired three sets of 360 evenly spaced radial spokes in the *x-y*, *x-z* and *y-z* plane, respectively, prior to the actual UTE measurement. All parameters of the calibration scan (e.g., gradient strength and timing, data sampling parameters) were identical to the actual *k*-space measurement to ensure that delays estimated from the calibration were compatible to the measurement data. However, during the initial dead time interval of the ADC (60 μs delay, see Fig 2A) a short dephasing gradient with maximum slew rate was switched on during the calibration scans. This short dephaser shifts the traversal of *k*-space center into the ramp up of the readout gradient. To estimate the gradient delays the time interval *t*_*A*_ between the start of the ADC and the traversal of *k*-space center was determined from the *k*-space phase information of the calibration data. Contrary to the magnitude data (Fig 3A), which exhibit a broad maxima that can hamper the extraction of it’s precise position, *k*-space phase data (Fig 3B) form a distinct fixed point (phase node) at the center of *k*-space at which all spins constituting the measurable magnetization reach phase coherence again. This holds true regardless of object phase or object shape.

To estimate the position of this phase node we calculated the deviation of all spokes from the mean phase, whereupon the point, at which the sum over all these deviations is minimal, indicates the phase node position on the sampling grid. To achieve sub-dwell time accuracy we performed a linear next neighbor fit between the sampling points surrounding the phase node (Fig 3C). From all *N* linear fit functions *ϕ*_*i*_ = *m*_*i*_ ⋅ *t* + *n*_*i*_ and the mean fit $\underline{\phi }=\underline{m}\cdot t+\underline{n}$ the sum of squared differences was calculated and minimized in order to obtain *t*_*A*_ analytically:

$$0=\frac{\partial }{\partial \text{t}}\sum_{\text{i}=1}^{360}{\left(\underline{\phi }-\phi_{\text{i}}\right)}^{2}$$ (1)

$${\text{t}}_{\text{A}}=-\frac{\sum_{\text{i}}^{360}(\underline{\text{m}}-{\text{m}}_{\text{i}})\cdot (\underline{\text{n}}-{\text{n}}_{\text{i}})}{\sum_{\text{i}}^{360}{\left(\underline{\text{m}}-{\text{m}}_{\text{i}}\right)}^{2}}$$ (2)

This calculation was performed for all three calibration planes and all coil channels, resulting in a mean time $\underline{t_{A}}$ of the actual *k*-space center crossing after averaging over all results. From the known gradient parameters the time *t*_*C*_ was calculated at which the readout gradient of the calibration should have compensated the dephaser and compared to the actual crossing of the *k*-space center $\underline{t_{A}}$ as extracted from the calibration scan. The resulting time delay $\Delta t=\underline{t_{A}}-t_{C}$ between gradient activity and sampling was then used for the correction of the actual measurement data.

### Image Reconstruction

To handle the large reconstruction matrix sizes and high amount of input data image reconstruction was performed off-scanner on a high performance computation system with multiple CPU cores (8 quad cores) and a total of 128 GB of memory using MATLAB (2010b, The MathWorks, Natick, MA). The 3d regridding was implemented in C++ with source code obtained from the MRI Pulse Sequence Design and Reconstruction Source Code Repository at http://www.ismrm.org/mri_unbound/sequence.htm [18].

To combine the radial spokes into a single Cartesian *k*-space state-of-the-art 3D gridding was performed using iterative sampling density correction [18]. To calculate 3D *k*-space coordinates for all data points, gradient integrals were calculated stepwise for each readout, taking into account the actual gradient parameters (amplitude and plateau, ramp up, ramp down times) as well as the delay *Δt* extracted from the calibration scan. The size of the target matrix for 3D gridding was increased by a grid scaling factor of 2 to reduce fold-over artifacts and side lobes.

In order to minimize memory demands during data processing the reconstruction first calculated the gridding weights based on the delay corrected k-space sampling positions and then subsequently applied those density weights during 3D gridding [18]. Then channel by channel the regridded data underwent 3D Fast-Fourier-Transformation after which the magnitude was squared and added to the final image matrix. After processing all channels the square root was taken of the image matrix.

### MR measurements

#### Phantom measurements

A structural phantom consisting of acrylic, Ni^2+^ doped water and rubber gaskets was scanned by using the vendor supplied 12-channel head matrix coil.

The phantom was scanned with the following common parameters for the single and dual echo UTE sequence: field of view (FoV) 220 mm x 220 mm x 220 mm, matrix size 256 x 256 x 256, 206221 radial readouts for full Nyquist condition, dwell time 2.1 μs, TE = 60 μs, RF pulse duration 20 μs. Both sequences were set to the highest possible bandwidth and the dual echo and single echo sequence were set to minimal TR = 3.2 ms and TR = 1.1 ms in combination with flip angles of α = 8° and α = 5°, respectively. The flip angles were chosen corresponding to the Ernst angle of the phantom’s *T*_*1*_ = 365 ± 20 ms relaxation time to allow a fair comparison of image signal to noise ratio (SNR).

To assess the impact of the short TR on SNR, the fast single-echo sequence was repeated with two and three repetitions, the latter matching the acquisition time of the dual echo sequence. SNR was determined by evaluating a small region of interest (ROI) in a homogeneous signal region of the phantom, using the ROI’s standard deviation as a measure for the noise.

To assess the accuracy of the delay correction the single-echo data set was reconstructed three times, once without using delay compensation, once applying the automatically extracted time delay *Δt* to all three axis and last by using the extracted time delay *Δt* with additional, manually optimized, axis dependent delay corrections.

#### *In vivo* measurements

A total of 19 scans were performed on 6 different volunteers imaging different anatomical regions and using different measurement parameters to evaluate the robustness and accuracy of the automatic delay estimation. The volunteer scans were approved by the local ethics committee and all volunteers gave their written consent.

To illustrate the potential of the optimized sequence different scan applications were selected. High resolution imaging data of the head were acquired with curvilinear reformatted slices along the sagittal suture of the skull and reformatted images of the denture which were created by using Syngo Fast View (Siemens Healthcare, Erlangen, Germany).

Images of the tibial cortical bone were acquired with isotropic resolution of (1 mm)^3^, repeated for TE = 60 μs / 110 μs / 190 μs / 400 μs / 2460 μs / 4920 μs and a common TR = 6 ms to accommodate the second in phase echo time. T_2_* maps were calculated using a state-of-the-art fitting algorithm with noise bias compensation [28]. For comparison an additional acquisition using the dual echo UTE sequence was also performed on the same subject with the same resolution and TR = 4 ms. The primary goal is to estimate the T_2_* values of cortical bone, hence the shortest possible time TE2 = 1370μs was chosen. T_2_* maps were calculated from these two echoes using the same fitting algorithm [28].

The fast single echo sequence was also applied for imaging lung tissue within a single breath hold using radial under-sampling. Parameters were FoV = 300 mm x 300 mm x 300 mm, matrix size 160 x 160 x 160, dwell time = 1.55 μs, TR = 0.9 ms, TE = 70 μs, 31058 radial readouts corresponding to 39% of full Nyquist sampling resulting in TA = 29 s. Reconstruction of the under-sampled data comprised gradient delay correction and regridding of the data as described before. Maximum intensity projections (MIPs) in the three orthogonal planes were calculated from the acquired 3D data set through a slice stack of 20 mm.

## Results

### Phantom measurements

**Fig 4:**
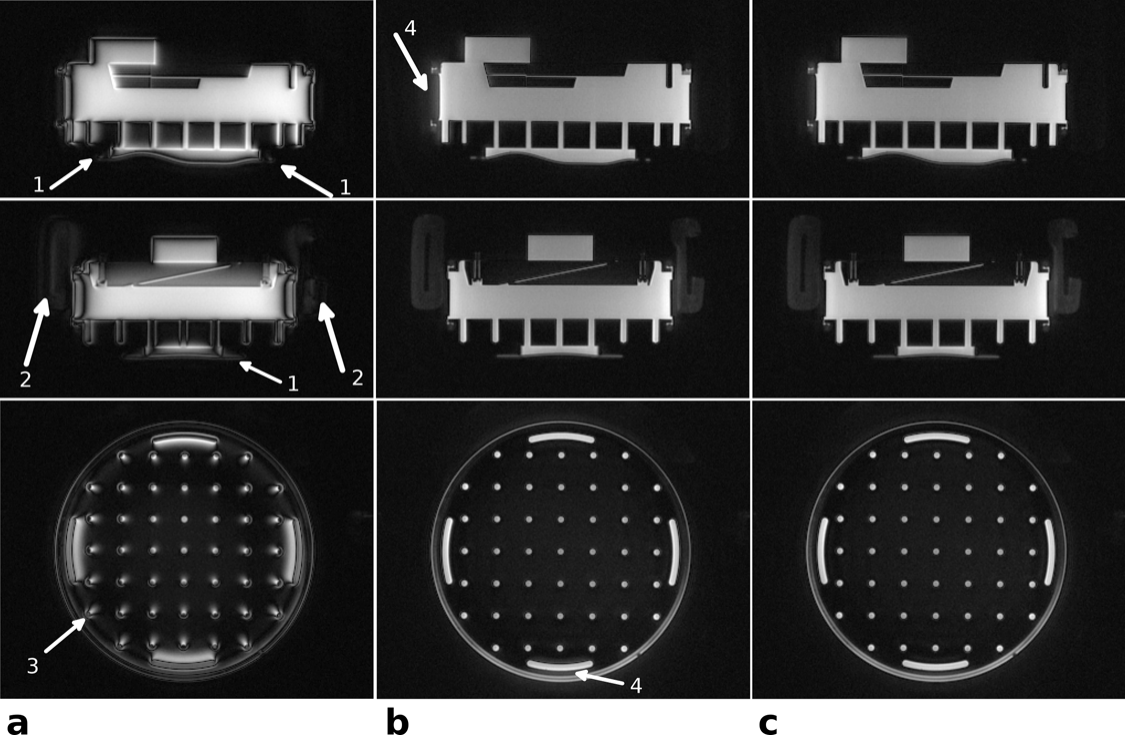
**Phantom images—the effect of delay correction:** Images in three orthogonal planes of a structure phantom reconstructed without gradient delay correction (a), with global delay correction as extracted from the template (b), and with both global delay and axis-specific delays (c). Without delay correction the visibility of the short T_2_ rubber gasket (arrows 1) and the restraining pads of the head coil (arrows 2) is strongly reduced (a). The strength of the delay effects depends on the distance from iso-center (see arrow 3), where the outermost structures show the strongest signal shift. The applied automatic global delay correction in (b) highly reduces these artifacts, but diffuse signal outside the object is still visible in some directions (arrows 4). These signal shifts can be further reduced by applying manual, axis specific delay corrections of, in this case, Δ*t*_*x*_ = +0.75 μs, Δ*t*_*y*_ = −0.1 μs and Δ*t*_*z*_ = 0.0 μs.

Fig 4 demonstrates the influence of the delay correction on image quality. The images in Fig 4A were reconstructed without gradient delay correction, resulting in distorted, shifted and blurred images as well as signal voids throughout the phantom. The observed signal shifts are stronger with increasing distance from the iso-center (see arrow 3).

The gradient delays were estimated from the acquired calibration scan to be *Δt* = 5.1 μs and the corrected images (Fig 4B) revealed only small residual artifacts that are only visible at the outermost structures (arrows 4). The additional correction of axis-specific gradient delays (*Δt*_*x*_ = 0.75 μ*s*, Δ*t*_*y*_ = −0.1 μ*s*, Δ*t*_*z*_ = 0.0 μ*s*) further reduced these residual delay artifacts (Fig 4C). The short *T*_*2*_ materials, like the rubber gasket (see arrows 1) and the restraining pad of the coil (see arrows 2) were barely visible without gradient delay correction but became well delineated with correction (Fig 4B and 4C).

### *In vivo* measurements

#### Head imaging

**Fig 5:**
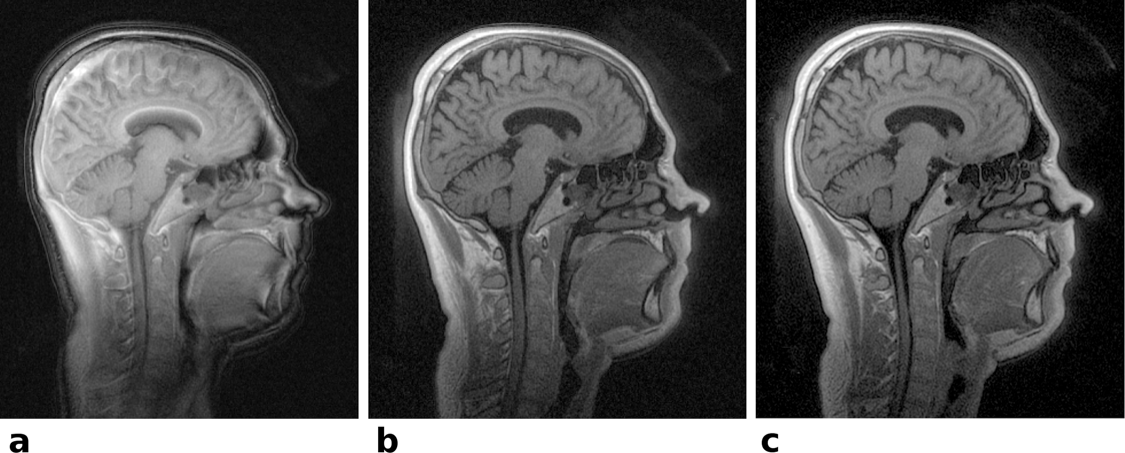
*In vivo* head images–the effect of delay correction: Central sagittal slices of a volunteer’s head. (a) The reconstructed image of the single echo sequence without delay correction exhibits severe signal shifts compared to the images corrected with the system delay obtained from the template scan (b). Note the large signal voids in the uncorrected image (a), especially at the outer edges, like the chin and the face area. The same slice acquired with the dual echo reference sequence and with delay correction is shown in (c). The spoiling regime applied to the single echo sequence does not cause any visible artifacts (b) compared to the dual echo sequence with constant spoiler moments (c).

**Fig 6:**
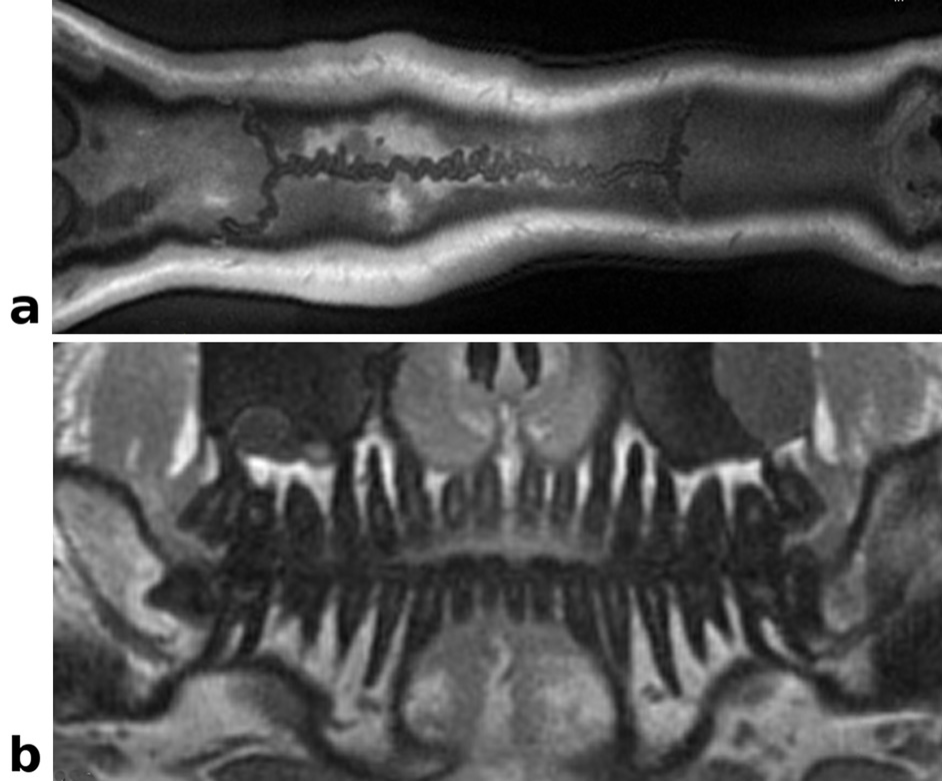
Curved reformatted *In vivo* head images: Re-sliced UTE images obtained from the same data set as in Fig 6. (a) Curved reformatted slice displaying the sagittal suture. (b) Curvilinear reformatted view of the denture, mimicking a dental panorama image. The nerve canals, dentin and the jawbone are clearly delineated.

Images acquired with the single echo UTE sequence without delay correction, suffering from image blur, signal shifts and signal voids at the outer edges, are shown in Fig 5A. In comparison the automatically delay corrected images with only minor residual delay artifacts are given in Fig 5B. Due to the short echo time and high isotropic resolution of (0.83 mm)^3^ even small anatomical structures in the nasal cavities are well delineated with almost no susceptibility caused distortions or signal voids. A reference image, acquired as first echo of the dual echo UTE sequence, is shown in Fig 5C and provides identical image quality with the exception of higher SNR due to the longer TR and acquisition time. Curvilinear reformatted images obtained from the same isotropic 3D data set as Fig 5B delineate the sagittal suture (Fig 6A) and the teeth (Fig 6B) very well.

#### Tibial cortical bone imaging

**Fig 7:**
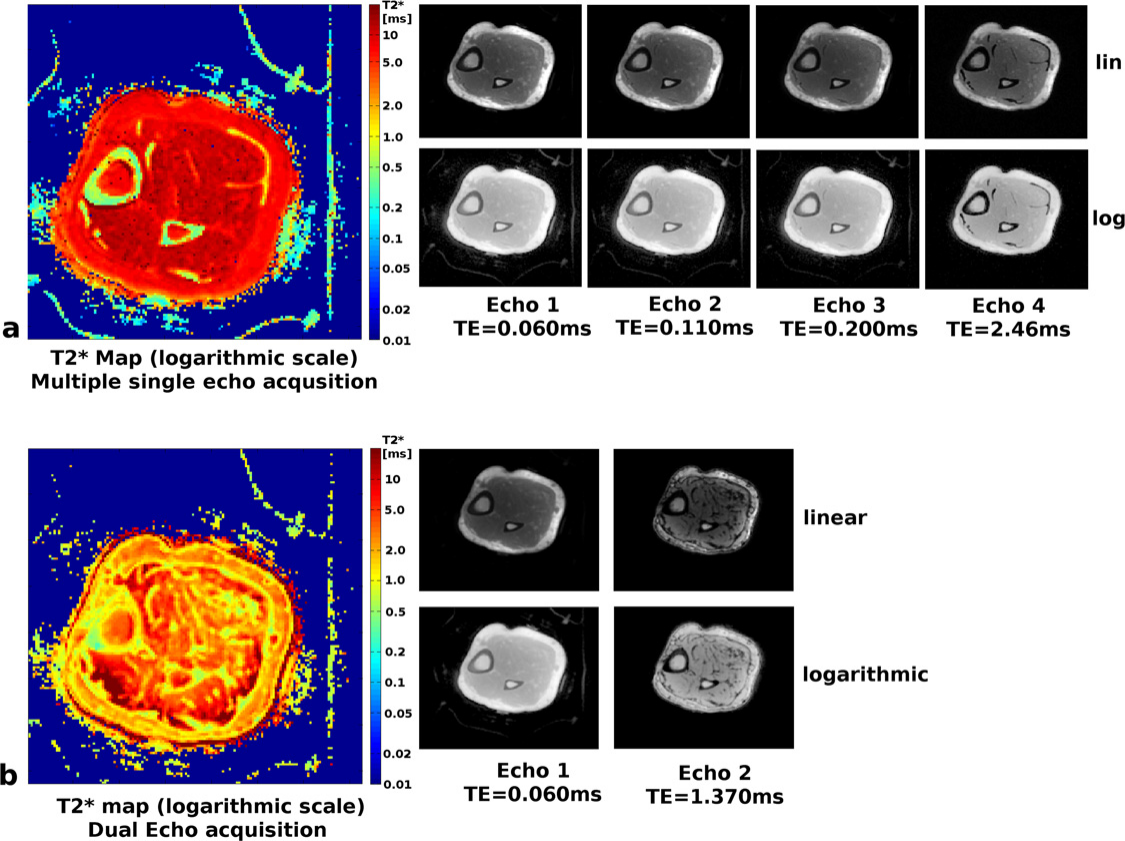
**UTE images and T**_2_* maps of the tibial cortical bone: (a) Single echo sequence with individual acquisitions of different echo times (TR = 6 ms). The individual images at each echo time are shown with linear scaling of the gray values in the top row and with logarithmic scaling in the bottom row. The T_2_* map on the left is also displayed with a logarithmic color scale in units of ms. (b) Single run of the dual echo sequence with the shortest possible second echo time TE = 1370 μs and TR = 4 ms. The top row shows linearly and the bottom row logarithmically scaled gray values. The T_2_* map on the left is again displayed with a logarithmic color scale.

Images and corresponding T_2_* maps of the tibial cortical bone are shown in Fig 7. Using the single echo acquisition with 3 short echo times resulted in T_2_* values of 0.59 ± 0.39 ms for cortical bone, revealing even small variations of T_2_* in different bone areas (Fig 7, top). The two longest echo times allowed accurate estimation of T_2_* for other tissues (muscle T_2_* = 10.9 ± 1.34 ms, fat T_2_* = 7.4 ± 0.89 ms). The two echo times of the dual echo sequence had the minimum possible echo spacing (TE2 = 1370 μs); exhibiting opposed-phase effects in the bone marrow, muscle and fat tissue and thereby underestimating the T_2_* values considerably (muscle T_2_* = 2.5 ± 1.4 ms, fat T_2_* = 2.4 ± 0.89 ms). The T_2_* values of cortical bone were, on the other hand, overestimated (T_2_* = 1.1 ± 0.68 ms) (Fig 7, bottom) due to the long second echo time.

#### Lung imaging

**Fig 8:**
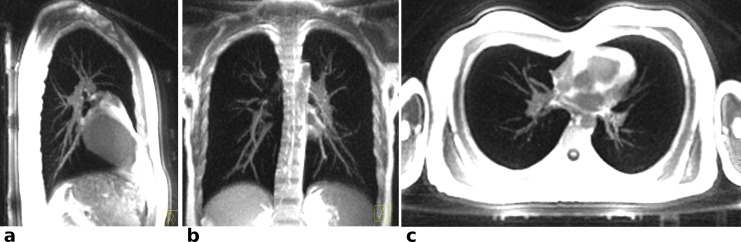
UTE single breath-hold lung imaging: Maximum intensity projections of UTE lung images over 20 mm of a healthy volunteer. The original images were acquired with an isotropic resolution of (1.8 mm)^3^ at 39% Nyquist sampling within a single breath hold of 29s. In (a) and (c) parts of the spine coil and the scanner table are seen. Due to the large FoV some image distortions are visible towards the edges of the images.

A complete thorax scan of a volunteer was acquired within a single breath-hold of 29 s and with isotropic resolution of (1.8 mm)^3^. Fig 8 shows maximum intensity projections (MIPs) delineating the lung’s internal structures, such as blood vessels and air paths. Animated MIPs in all three cardinal directions are available as S1–S3 Figs. These lung images, particular around the heart, also demonstrate very clearly the absence of artifact bands (ghosting in phase direction), which are caused by motion in Cartesian sampling. Instead, without applying cardiac gating, motion causes slightly blurred images of the heart and the aorta. The under-sampling of the data caused minor streaking artifacts which, however, mostly manifested outside the volunteer’s body and did not degrade image quality in the body parts of interest.

#### Robustness of the gradient delay estimation

Table 1 summarizes the acquisition parameters and investigated anatomical regions as well as the obtained parameters for delay correction for the 19 volunteer scans acquired within a wide range of acquisition parameters. The automatic gradient delay estimation was in all cases close to the manually determined optimal value with a mean difference of −0.13 ± 0.45 μs, and never exceeded ±1 μs. The resulting images showed only small differences and signal shifts between the automatically and manually determined gradient delays comparable to Fig 4B and 4C.

**Table 1:** Overview of scan parameters and delay correction for volunteer exams.

Organ	FoV (mm³)	Matrix	TE (μs)	Dwell time (μs)	Reconstruction time	Automatic delay (μs)	Manual delay correction (μs)
Abdom	300	256	60	1.55	01:41:16	7.37	1.0
Head	220	128	60	2.10	00:14:05	5.15	0.0
Head	200	128	100	2.30	00:08:26	5.36	0.0
Head	208	208	60	2.25	00:48:16	7.45	0.0
Head	208	240	60	2.20	01:08:53	4.42	0.0
Head	220	256	60	2.20	01:17:15	4.71	0.5
Head	200	240	60	1.53	01:26:33	5.25	0.0
Head	208	256	60	2.20	01:34:00	3.69	0.5
Head	200	256	60	2.30	01:13:44	4.17	-0.5
Head	200	288	60	2.30	02:08:04	4.43	0.0
Lung	280	144	60	1.65	00:10:40	6.85	-1.0
Lung	300	160	60	1.55	00:15:09	7.87	-1.0
Lung	280	176	60	1.65	00:31:00	7.28	-1.0
Lung	300	192	60	1.55	00:58:40	5.17	0.5
Lung	300	208	60	1.55	00:52:02	7.88	-1.0
Foot	200	240	60	2.45	01:28:02	7.93	0.0
Knee	180	208	100	2.55	00:37:23	8.77	-0.5
Tibia	208	208	60	2.20	00:49:52	3.68	0.0
Tibia	144	144	60	2.10	00:06:20	5.44	0.0

The table summarizes the UTE sequence parameters used for imaging different body parts and organs of volunteers. The resulting reconstruction times (hh:mm:ss) were determined from a single reconstruction run on a computer cluster and are only rough estimates. The optimum delay is the automatically determined delay plus the manual delay correction.

## Discussion and Conclusion

3D radial center-out acquisition provide several advantages over Cartesian trajectories, allowing much shorter echo times, and, since 3D radial acquisition averages over inner *k*-space, motion artifacts are reduced. Furthermore, the readout oversampling which is intrinsically applied in all spatial directions renders the reconstructed images very robust against fold-over artifacts.

Radial 3D trajectories are often considered as inefficient, especially in comparison to Cartesian sampling due to the larger number of readouts required for a fully Nyquist sampled data set. However, the presented UTE modification reduces the time of a single readout and allows for *TR* as short as approximately 1 ms. Consequently, fully Nyquist sampled 3D radial data can, in practice, be collected within an acquisition time that is comparable to a Cartesian acquisition with the added benefit of fold-over protection and motion artifact reduction. One further important aspect concerns the possibility of accelerating data acquisition: With Cartesian sampling and current linear acceleration methods [29, 30] this possibility is inherently limited, leading to speed-up factors of typically 2 with conventional Cartesian under-sampling, i.e., partial Fourier. Parallel imaging acceleration is limited by *g*-factor noise [31–33] and non-linear acceleration techniques, like compressed sensing [34–36], have been shown to work well only with randomized Cartesian sampling [34, 37, 38]. Equidistant Cartesian trajectories are not optimal for the latter method [36, 39]. In contrast, radial acquisitions can be combined very well with compressed sensing reconstruction techniques [34, 36, 40, 41] since the radial projections intrinsically distribute any aliasing artifacts into a diffuse background [36]. In effect 3D radial acquisitions, in particular, are well suited for under-sampling [42, 43] and combinations of acceleration methods allowing promising future improvements of image quality and scan speed [44].

One challenge with radial 3D center-out acquisitions, however, are hardware inaccuracies and unwanted side effects, like eddy currents, as well as scan parameter dependent jitter that can introduce delays between the sampling time points and the gradient timing [16, 17]. With radial sampling trajectories these delays cause incoherent shifts of the sampling coordinates and, particularly in combination with ramp sampling, may cause severe image artifacts as illustrated in the uncorrected images (Figs 4A and 5A). As described in [16] there may be constant hardware delays associated with each gradient axis, which for the MR scanner used in the present work were less than 1 μs. Larger additional delays, which are constant on all three gradient axes, can be caused by gradient raster timings and jitter effects and depend on sequence parameters, like bandwidth, matrix and field-of-view. We observed these delays to be in the range of typically 3–7 μs. Our presented solution employs quick template scans to estimate these axis independent gradient delays robustly and reproducibly for a variety of objects ranging from compact phantoms to imaging head and lung *in vivo*. In contrast to other methods, e.g. measuring the actual trajectory shape, the proposed estimation is faster and does not depend in any way on a known object shape or positioning slices inside the object. The proposed calibration performs very robust as long as there is some signal generating object within the scanner. This hold even true if only short T_2_ materials are in the scanner, since the calibration scan itself is a UTE acquisition.

Although axis dependent gradient delays certainly exist [16], the proposed calibration provided very good image quality with only minor residual delay artifacts being visible in the phantom (Fig 4B) and in vivo head images (Fig 5B). One reason for the relatively low impact of axis dependent gradient delays on image quality is most likely due to the fact that all readouts start directly in the center of *k*-space, oversampling the correct k_0_. In contrast, for non center-out radial sequences, gradient delays may cause the readout trajectory to actually miss the center of *k*-space [16], leading to more severe artifacts.

Comparing image quality and scan time between the two presented sequence variants the first echo, acquired with the dual echo sequence, clearly provides the best SNR with excellent image sharpness and details (Fig 5C). Furthermore, the dual echo sequence has a constant and direction independent spoiling moment for each TR, which is well understood and achieves predictable spoiling effectiveness comparable to Cartesian spoiled gradient echo sequences. While the dual echo sequence enables suppression of tissues with long T_2_ relaxation times [21, 45] and T_2_* mapping, the choice of the second echo time is very limited. The second echo is nominally acquired when the trajectory gets back to *k*-space center, which leads to TE2 > 1ms. T2*-mapping of short T_2_-components requires sufficiently short echo times for both TE1 and TE2 or the results will be biased towards longer T_2_* values.

The presented single-echo UTE sequence provides factor 3 faster acquisition time compared to the dual echo variant without any spoiling artifacts. The lower SNR of the single-echo sequence can be entirely attributed to the reduced scan time, indicating the absence of additional noise due to incoherently refocused magnetization and confirming the effectiveness of the spoiling. For long T2 tissue suppression other methods than echo subtraction can be employed [46, 47].

Quantitative T_2_* maps can be calculated from repeated single echo acquisitions with different echo times that can be freely adjusted to match the relaxation time of interest. With three echoes ranging from 60 μs to 200 μs accurate T_2_* mapping of, e.g. cortical bone in the tibia was possible (Fig 7A). If cortical bone characterization is the sole aim, this can be accomplished with a common TR of 1.4ms, requiring for all three runs roughly the same acquisition time as one conventional dual echo acquisition. Not only does the fast single echo provide three fitting points instead of only two, the free choice of TE allows optimal coverage of the signal decay curve. In contrast the dual echo sequence can only provide a second echo at TE2 = 1.37ms, which is much too late for accurate cortical bone T2* mapping (Fig 8B).

In conclusion, we have presented a fast radial 3D sequence on a 3 T clinical scanner that provides ultra-short echo time acquisition (TE ~ 60 μs) with much shorter acquisition time than comparable dual echo UTE sequences. The implemented gradient delay calibration requires approximately 1s scan time and does not need any user interaction. Calculation of the gradient delay is based on the analysis of the *k*-space phase of the readout data, which works very robustly in phantoms and *in vivo* for very different anatomical regions. The implemented variable readout length spoiler minimizes the time required for spoiling and allows very short repetition times of 1 ms or shorter, facilitating clinically acceptable acquisition times of a few minutes which can be even further reduced to a single breath-hold by applying radial under-sampling.

## Supporting Information

S1 Fig Maximum intensity projections of UTE lung images over 20 mm of a healthy volunteer (same data as Fig 8) as animated flythrough reconstructed in coronal view. The original images were acquired with an isotropic resolution of (1.8 mm)^3^ at 39% Nyquist sampling within a single breath hold of 29s.
(AVI)

S2 Fig Maximum intensity projections of UTE lung images over 20 mm of a healthy volunteer (same data as Fig 8) as animated flythrough reconstructed in sagittal view. The original images were acquired with an isotropic resolution of (1.8 mm)^3^ at 39% Nyquist sampling within a single breath hold of 29s.
(AVI)

S3 Fig Maximum intensity projections of UTE lung images over 20 mm of a healthy volunteer (same data as Fig 8) as animated flythrough reconstructed in axial view. The original images were acquired with an isotropic resolution of (1.8 mm)^3^ at 39% Nyquist sampling within a single breath hold of 29s.
(AVI)
